# Evaluation of miR-506 and miR-4316 expression in early and non-invasive diagnosis of colorectal cancer

**DOI:** 10.1007/s00384-017-2814-8

**Published:** 2017-04-12

**Authors:** Paweł Krawczyk, Tomasz Powrózek, Tomasz Olesiński, Adam Dmitruk, Joanna Dziwota, Dariusz Kowalski, Janusz Milanowski

**Affiliations:** 1 Department of Pneumonology, Oncology and Allergology, Medical University of Lublin, Jaczewskiego 8, 20-954 Lublin, Poland; 2 Department of Oncological Gastroenterology, Maria Skłodowska-Curie Memorial Cancer Center and Institute of Oncology in Warsaw, WK Roentgen 5, 02-781 Warsaw, Poland; 3 Department of Lung and Chest Tumor, Maria Skłodowska-Curie Memorial Cancer Center and Institute of Oncology in Warsaw, WK Roentgen 5, 02-781 Warsaw, Poland

**Keywords:** Colorectal cancer, Early diagnosis and prevention, microRNAs, Liquid biopsy

## Abstract

**Purpose:**

Examination of the entire colon by colonoscopy remains the golden standard for screening of colorectal cancer (CRC). However, patients are reluctant to perform invasive colonoscopies because of interference with their intimacy. Therefore, the potential use of non-invasive analysis of microRNAs expression in liquid biopsy as a novel biomarker for early CRC has investigated in several studies. In this study, we analyzed the expression of two novel microRNAs: miR-506 and miR-4316, which have never been examined in CRC.

**Methods:**

Plasma samples were collected from 56 patients (median age of 68 years) with operable colorectal cancer and from 70 healthy individuals (median age of 59 years). Expression of plasma microRNAs was evaluated by quantitative reverse transcription polymerase chain reaction using Eco real-time PCR device (Illumina, USA).

**Results:**

We found a significant elevated expression of both examined microRNAs in early CRC patients when compared to those in healthy individuals (*p* = 0.0054 for miR-506 and *p* = 0.0025 for miR-4316). The expression of miR-506 and miR-4316 did not depend on gender, age, disease stage, and tumor localization of CRC patients. ROC curve analysis showed that both examined microRNAs could differentiate early stage colorectal cancer from healthy individuals with 76.8% specificity and 60.7% sensitivity for miR-506 analysis and 76.8% specificity and 75% specificity for miR-4316 analysis.

**Conclusion:**

Our study revealed that elevated expression of miR-506 and miR-4316 in peripheral blood were potential molecular markers for early colorectal cancer.

## Introduction

Colorectal cancer (CRC) is the third most commonly diagnosed cancer in males and the second in females. In the developed countries, the CRC incidence and mortality have been slowly but steadily decreasing due to increased endoscopic screening and efficacy of surgical treatment. Resection of early stages CRC allows recovery almost 100% of patients. However, many patients avoid prophylactic examinations because of fears prior to colonoscopy and violation of privacy during this procedure. Therefore, it is necessary to search a low-cost, non-invasive screening method with high sensitivity and specificity to CRC detection.

Determination of epigenetic events may be a useful tool for early and non-invasive (tests performed in peripheral blood) cancers detection. Gene expression regulation by aberrant DNA methylation and by microRNAs is a well-characterized event in tumor biology and is extensively described for CRC. CRC patients are the first group of cancer patients who may benefit of a epigenetic diagnostic test. Increased levels of cell-free circulating, methylated promoter region of *Septin 9* gene have been reported in CRC patients. Unfortunately, the sensitivity of this test is insufficient in early stages of CRC (stage I 52%, stage II 76%, stage III 76%). Moreover, the organ specificity of the test is low. The presence of *Septin* 9 gene methylation in free-circulating DNA (cf-DNA) was detected in the plasma of patients with other types of cancers, e.g., lung cancer (LC) [1].

Currently, the potential use of microRNAs analysis as a novel biomarker for cancers has investigated in several studies (including measuring of microRNAs expression from disintegrated tumors cells in liquid biopsy). microRNAs are class of small, non-coding RNA molecules that regulate gene expression by inducing mRNA degradation or suppressing translation. microRNAs are usually complementary to the short nucleotide sequences in the 3’UTR (untranslated region) of one or more mRNAs. Abnormal expression of microRNAs specific for mRNA of tumor suppressor genes and oncogenes is showed in many cancer types and appears to be cell type and disease specific [2].

Here, we analyzed the expression of two novel microRNAs: miR-506 and miR-4316, which have never been investigated in CRC. miR-505 targets 3’UTRs of *GATA6* gene for GATA6 transcriptional factor, *YAP1* gene encoding a transcriptional co-activator, *FOXQ1* oncogene, EMT (epithelial-mesenchymal transition)-related genes such as *SNAI2*, *VIM*, and *CD151* as well as gene for sphingosine kinase 1 (*SPHK1*) thereby inhibiting Akt/NF-κB signaling [3–8]. miR-4316 targets has not been clearly identified. However, study of Goff and co-workers suggested that miR-4316 can be complementary to the 3’UTR of mRNA *AGO2* gene. Argonaute (AGO1-4) proteins themselves are involved in the silencing of gene expression with the participation of microRNAs. Argonaute selects the single guide strand of the mature microRNAs to load into the RISC (RNA-induced silencing complex). This single strand microRNA acts as a template for RISC to recognize complementary mRNA transcript [8–10].

## Material and methods

Our study enrolled 56 chemotherapy and radiotherapy naïve CRC patients (median age of 68 years, 37 males, 17 females) with operable colorectal cancer (42.9% in first and 57.1% in second stage of the disease). Material was obtained before application of surgical procedures. Additionally, plasma samples were collected from 70 healthy individuals (median age of 59 years, 39 males, 31 females) without gastrointestinal disorders and cancers history.

Blood samples were collected into tubes covered with EDTA-K2. Samples were stored at room temperature for a few minutes and centrifuged at 3000 rpm for 10 min. At least 2 ml of plasma were stored in cryovials at −80 °C.

Total RNA including small non-coding RNAs (<100 nucleotides) was extracted from 400 μl of plasma using miRNeasy serum/plasma kit (QIAGEN, USA) according to the manufacturer’s protocol. Quantitative reverse transcription polymerase chain reaction (qRT-PCR) was conducted in two steps. Firstly, miRNAs were reverse transcribed to complementary DNA (cDNA) using MicroRNA Reverse Transcription Kit with specific RT primers, which copied only selected microRNAs (Applied Biosystems, USA). Reverse transcription was performed in TPersonal Thermocycler (Biometra, Germany). In the second step, cDNA was quantified using TaqMan Universal Master Mix II with Uracil N-glycosylase (UNG) and TaqMan microRNA-specific primers with fluorescently labeled probes (Applied Biosystems, USA) in Eco real-time PCR device (Illumina, USA). microRNAs expression was normalized relative to U6 small nuclear RNA (U6 snRNA). The ΔCt, 2^−ΔCt^ and 2^−ΔΔCt^, analysis was used to compare microRNAs expression between CRC patients and healthy individuals.

MedCalc Software version 12 (Belgium) was used for statistical analysis. Relative expression of microRNAs between studied groups was compared using *t* test. Fisher’s exact test was used to compare the groups of CRC patients with different clinical characteristics as well as microRNAs expression (calculated as “high” if the expression was equal or higher than the median). For assessment of diagnostic accuracy (test sensitivity and specificity), the receiver operating curves (ROC) with area under curve (AUC) analysis were generated. *p* values of <0.05 were assessed as statistically significant.

The study was approved by the local Ethical Committee of Medical University of Lublin (KE-0254/218/2015).

## Results

We found a significant elevated expression of both examined microRNAs in CRC patients when compared to those in healthy individuals (*p* = 0.0054 for miR-506 and *p* = 0.0025 for miR-4316). These differences were independent of the CRC stage. The expression of analyzed microRNAs in patients with stage I of CRC differed significantly from healthy individuals (*p* = 0.0032 for miR-506 and *p* = 0.0005 for miR-4316). Similar differences were observed between patients in stage II of CRC and healthy individuals (*p* = 0.0319 for miR-506 and *p* = 0.0016 for miR-4316). However, expression of both microRNAs did not differ significantly between patients in stage I and II of CRC. The high expression of miR-506 was significantly more frequently observed in non-smoking CRC patients than in smoking CRC patients (*p* = 0.029). The expression of miR-506 and miR-4316 did not depend on gender, age, and tumor localization.

ROC curve analysis showed that both microRNAs could differentiate early stage CRC from normal controls with an AUC of 0.747 for miR-506 (95% CI 0.662–0.820) and 0.744 for miR-4316 (95% CI 0.658–0.817). For miR-506 analysis, the specificity of the test was 76.8% and sensitivity was 60.7%. For miR-4316 analysis, the specificity and the sensitivity were 60.9 and 83.9%, respectively. Combination ROC analysis of both microRNAs expression revealed a litter increased AUC value of 0.751 (95% CI 0.666–0.8240) with 76.8 and 75% specificity.

## Discussion

Several studies have examined microRNAs expression in CRC and confirmed that this expression is altered in CRC. This indicates that microRNAs may have more oncogenic than tumor suppressive functions in CRC [11]. In contrast to CRC, microRNAs expression are generally reduced in other types of cancers. These observations have been made in the material derived from tumor tissue compared to surrounding normal tissue [2, 11]. Therefore, microRNAs expression patterns could classify tissue and tumor types [2]. However, the examination of microRNAs expression in tumor tissue could not be used for CRC early diagnosis. Therefore, circulating microRNAs detected in liquid biopsy are being evaluated for potential marker in early CRC detection [12].

Our analysis revealed that the elevated expression of miR-506 and miR-4316 in peripheral blood met the above assumptions. These microRNAs were not previously investigated in CRC patients and in the liquid biopsy. In our previous study, we confirmed that expression of miR-506 in liquid biopsy was significantly lower in LC patients compared to healthy individuals. Moreover, expression of miR-4316 in peripheral blood was similar in LC patients and healthy persons [8]. These observations may indicate applicability of the analysis of the expression of miR-506 and miR-4316 in distinction of CRC patients from LC patients and from healthy individuals.

Li et al. found that miR-506 expression was significantly lower in pancreatic cancer (PC) tissue compared to normal pancreas. High expression of miR-506 was good prognostic factor for PC patients. Authors concluded that transcriptional repression of miR-506 results in the upregulation of SPHK1, which has an important role in PC progression by regulating AKT/NF-κB pathways [7].

Zhang et al. revealed that expression of miR-506 was significantly lower in nasopharyngeal carcinoma (NPC) tissue and cell lines compared with normal nasopharyngeal specimens. miR-506 overexpression inhibited NPC cell proliferation and colony formations as well as repressed NPC cells invasion. Authors suggested that tumor suppressor role of miR-506 was mediated by FOXQ1 downregulation [5].

Breast cancer patients with high miR-506 expression in tumor tissue showed significantly longer distant relapse free survival compared to patients with low miR-506 expression. Arora et al. supposed that this phenomenon is associated with regulation by miR-506 the expression of EMT-related genes. In breast cancer cell lines, authors showed that the expression of CD151, VIM, and SNAI2 were suppressed, while CDH1 (an epithelial marker) was upregulated by miR-506 [6].

**Fig. 1:**
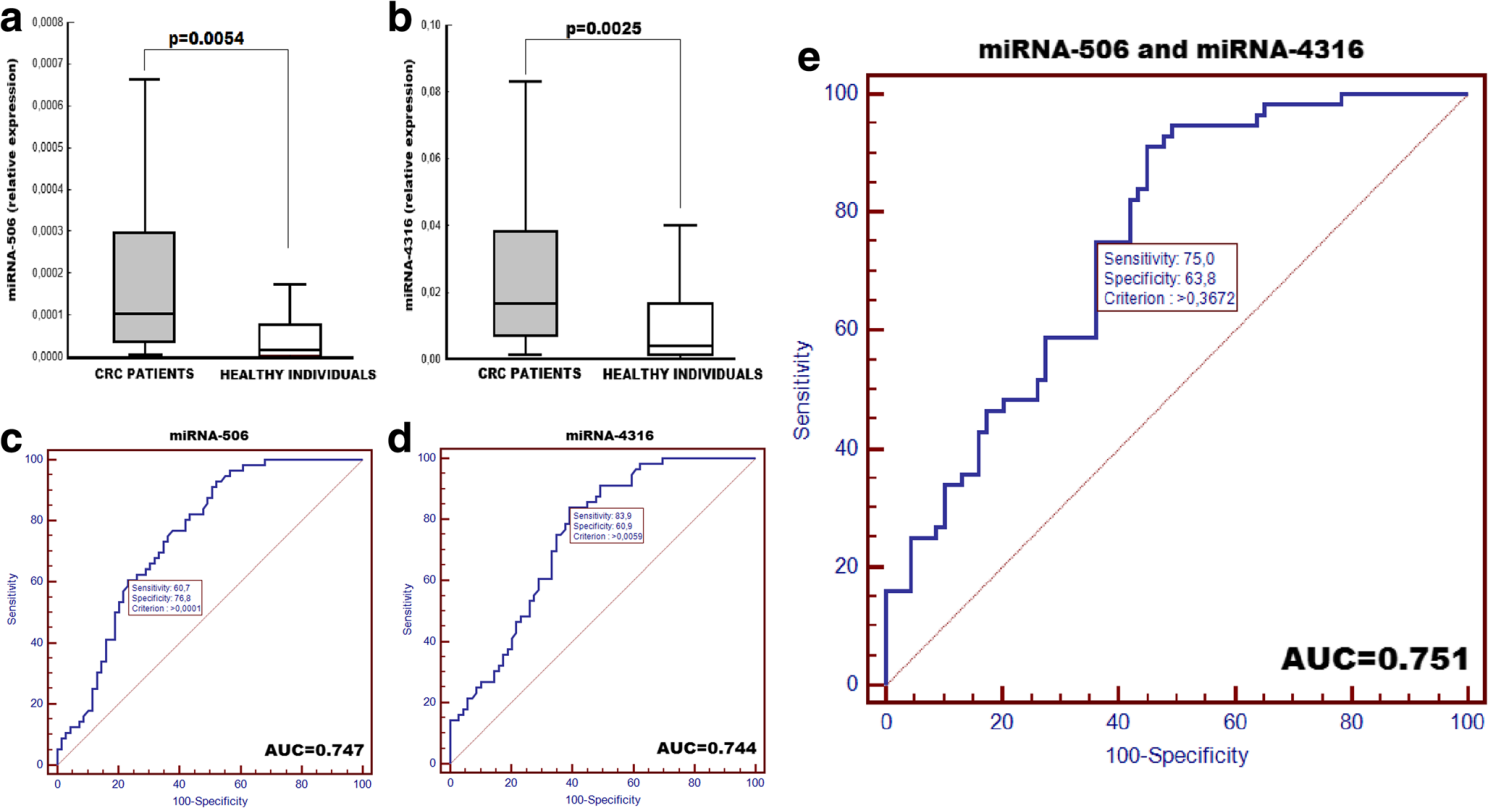
Comparison of miR-506 (**a**) and miR-4316 (**b**) expression in CRC patients and in healthy individuals. Receiver operating characteristic (ROC) curve analysis using examined miRNAs for discriminating colorectal tumors. AUC (the area under the ROC curve) estimation for the examination of miR-506 (**c**) and miR-4316 (**d**) as well as both miRNAs (**e**) in discrimination of the CRC patients from the healthy individuals

Taking into account the above facts, it is reasonable to investigate the possibility of application of miR-506 analysis in peripheral blood as a specific CRC marker. It appears that the increased miR-506 expression is characteristic for CRC, while in other types of neoplasms (lung, breast, NPC, and pancreatic cancer), the miR-506 expression could be reduced. Examination of miR-4316 expression in peripheral blood and tumor tissue of patients with different types of neoplasms requires further studies (Fig. 1.
